# Reorganization of Substance Use Treatment and Harm Reduction Services During the COVID-19 Pandemic: A Global Survey

**DOI:** 10.3389/fpsyt.2021.639393

**Published:** 2021-04-29

**Authors:** Seyed Ramin Radfar, Cornelis A. J. De Jong, Ali Farhoudian, Mohsen Ebrahimi, Parnian Rafei, Mehrnoosh Vahidi, Masud Yunesian, Christos Kouimtsidis, Shalini Arunogiri, Omid Massah, Abbas Deylamizadeh, Kathleen T. Brady, Anja Busse, ISAM-PPIG Global Survey Consortium, Adrian Octavian Abagiu, Marc N. Potenza, Hamed Ekhtiari, Alexander Mario Baldacchino

**Affiliations:** ^1^Department of Neuroscience and Addiction, School of Advanced Technologies in Medicine (SATiM), Tehran University of Medical Sciences, Tehran, Iran; ^2^Integrated Substance Abuse Programs Department, University of California, Los Angeles, Los Angeles, CA, United States; ^3^Behavioral Science Institute Radboud University, Nijmegen, Netherlands; ^4^Department of Psychiatry, Tehran University of Medical Sciences, Tehran, Iran; ^5^Materials and Energy Research Center, Tehran, Iran; ^6^Iranian National Center for Addiction Studies, Tehran University of Medical Sciences, Tehran, Iran; ^7^Department of Psychology, Faculty of Psychology and Education, University of Tehran, Tehran, Iran; ^8^School of Public Health, Tehran University of Medical Sciences, Tehran, Iran; ^9^Surrey and Borders Partnership NHS Foundation Trust, Leatherhead, United Kingdom; ^10^Turning Point, Eastern Health, Box Hill, VIC, Australia; ^11^Substance Abuse and Dependence Research Center, University of Social Welfare and Rehabilitation Sciences, Tehran, Iran; ^12^Rebirth Charity Society NGO, Tehran, Iran; ^13^Medical University of South Carolina, Charleston, SC, United States; ^14^Staff Member of Prevention, Treatment and Rehabilitation Section, Drug Prevention and Health Branch, Division for Operations, United Nations Office on Drugs and Crime, Vienna, Austria; ^15^Connecticut Council on Problem Gambling and Connecticut Mental Health Center, Yale School of Medicine, New Haven, CT, United States; ^16^Laureate Institute for Brain Research, Tulsa, OK, United States; ^17^School of Medicine, University of St Andrews, St Andrews, United Kingdom

**Keywords:** COVID-19 pandemic, substance use disorder, public health, drug policy, opioid agonist treatment, addiction services, harm reduction

## Abstract

**Background:** The coronavirus disease 2019 (COVID-19) pandemic has impacted people with substance use disorders (SUDs) worldwide, and healthcare systems have reorganized their services in response to the pandemic.

**Methods:** One week after the announcement of the COVID-19 as a pandemic, in a global survey, 177 addiction medicine professionals described COVID-19-related health responses in their own 77 countries in terms of SUD treatment and harm reduction services. The health responses were categorized around (1) managerial measures and systems, (2) logistics, (3) service providers, and (4) vulnerable groups.

**Results:** Respondents from over 88% of countries reported that core medical and psychiatric care for SUDs had continued; however, only 56% of countries reported having had any business continuity plan, and 37.5% of countries reported shortages of methadone or buprenorphine supplies. Participants of 41% of countries reported partial discontinuation of harm-reduction services such as needle and syringe programs and condom distribution. Fifty-seven percent of overdose prevention interventions and 81% of outreach services were also negatively impacted.

**Conclusions:** Participants reported that SUD treatment and harm-reduction services had been significantly impacted globally early during the COVID-19 pandemic. Based on our findings, we highlight several issues and complications resulting from the pandemic concerning people with SUDs that should be tackled more efficiently during the future waves or similar pandemics. The issues and potential strategies comprise the following: (1) helping policymakers to generate business continuity plans, (2) maintaining the use of evidence-based interventions for people with SUDs, (3) being prepared for adequate medication supplies, (4) integrating harm reduction programs with other treatment modalities, and (5) having specific considerations for vulnerable groups such as immigrants and refugees.

## Introduction

Coronavirus disease 2019 (COVID-19) was announced as a pandemic by the World Health Organization (WHO) on March 11, 2020 (1). COVID-19 quickly became a global concern given the rapid transmission of severe acute respiratory syndrome coronavirus 2 (SARS-CoV-2) (the infectious agent), lack of a vaccine or evidence-based treatments, person-to-person airborne spread of SARS-CoV-2, and high mortality of COVID-19 in specific populations, especially marginalized groups and/or those with preexisting conditions (2). Lack of capacity to anticipate, cope with, resist, and recover from COVID-19-related health consequences are related to individual vulnerability (3). To manage the current situation successfully, vulnerable groups should be recognized and helped with special considerations by relevant health systems (4).

According to the World Drug Report 2020, among ~269 million people with past-year drug use, over 35 million people experienced substance use disorders (SUDs) (5). People with SUDs (PWSUDs) may be particularly vulnerable to COVID-19 and its complications for multiple reasons (6). PWSUDs are at a higher risk of psychiatric problems such as mixed affective states (7); vice versa, polysubstance use and alcohol use disorder are common among patients with bipolar disorders (8). Moreover, PWSUDs experience underlying diseases that constitute risk factors for COVID-19 infection or can be exacerbated by it; for instance, long-term use of substances may cause cardiovascular problems (9) and chronic obstructive pulmonary disease (10). Such comorbidities may exacerbate superimposed COVID-19 symptoms and lead to higher mortality rates (11). Poor immune system functioning is also prevalent in PWSUDs because of chronic alcohol and drug use and blood-borne or sexually transmitted illnesses (12), poor nutritional status (13), and socioeconomic factors (14). Among PWSUDs, people who inject drugs (PWIDs) are at exceptionally high risk of COVID-19, as well as overdoses, unsafe injections, and risky sex (15).

Psychological conditions (e.g., phobia, anxiety, and panic attacks) during natural disasters and pandemics, which may be precipitated, perpetuated, or exacerbated through social isolation and quarantine, may lead at-risk people to start and/or relapse into drug taking (11, 16). Psychiatric comorbidity has a negative impact on recovery from COVID-19 and may increase the risk of non-fatal and fatal overdoses and suicides (16). In the general population, COVID-19 and related concerns such as potential mortality may act as internal stressors (17) and promote cognitive impairments (18) in domains such as decision making (19), problem solving (20), and attention (21) and thus may increase the incidence and prevalence of psychiatric disorders including PWSUDs (22).

Stigma may undermine social cohesion, contributing to situations in which the virus is more, not less, likely to spread. Such spread may result in more severe health problems and difficulties controlling a disease outbreak (23). There is an elevated likelihood for PWSUDs to be homeless and live in crowded shelters and neighborhoods (24). Synergistically, poor economic status linked to limited access to health care (25) may exacerbate risks for PWSUDs and PWIDs (15). Drug supply chains may be disrupted, and changes in licit and illicit markets may be accompanied by reductions in quality and safety (5, 26).

Furthermore, patients' accessibility to treatment services could be restricted due to lockdown policies (27). Patients receiving opioid agonist treatment (OAT) may not be able to access daily doses of medications (11); spatial distancing may make home detoxification difficult; and closing of non-essential services and utilizing staff and other resources to manage acute COVID-19 cases could result in sudden and uncoordinated closures of services for PWSUDs (26). Individuals who use multiple substances may be particularly impacted (28). Adaptive capacities of systems to epidemic situations that need coordinated responses may relate directly to vulnerabilities of the same systems (29). Accessibility to and equal distribution of wealth (financial and other resources, reliable and correct information and communication channels, appropriate and proportionate working technologies) compounded by reductions in social and relationship capital may impact social resilience to coping with pandemics (30).

To understand better complexities that are challenging addiction treatment and harm reduction services globally, the International Society of Addiction Medicine (ISAM) has been conducting a global longitudinal survey aiming to evaluate rapidly and over time how different countries are maintaining and/or reorganizing their substance use treatment and harm reduction services during the COVID-19 pandemic. This paper will report how different countries have adapted their health system response to emerging needs in the first month after the WHO's official announcement of the pandemic.

## Methods

Description of the methodology used for this survey has been published as a study protocol (31). Potential respondents were contacted on April 4, 2020 asking about the COVID-19 pandemic impact on PWSUDs in their own countries. Data collection was concluded on May 8, 2020.

### Questionnaire

The questionnaire consisted of 92 questions in two main areas: (1) situation assessment during the pandemic and (2) health responses to the pandemic. This paper will focus on health responses during the COVID-19 pandemic period (31). Results on the situation assessment are reported in another publication (32).

Questions around health responses to the pandemic were grouped into three categories:

(1) systems available to respond to acute emerging needs due to the COVID-19 pandemic within substance use services;
(2) availability of protocol and/or guidelines around COVID-19 and PWSUDs, and
(3) reduction in face-to-face contacts because of lockdown policies.

To assess respondents' overall views, they were asked to score the “overall situation at a glance” rating scale questions (RSQ) (between 1 and 10 with 1 for the worst situation and 10 for the best situation) based on their opinion regarding the overall quality of the situation of their country for each of the above three sections.

### Categorization of Countries Based on Their Income

The 2019 statistical annex of World Economic Situation and Prospects (WESP) (33) was used to categorize responding countries. Very low- and low-income categories were merged into one, retaining middle- and upper-income countries designations. In figures, countries' names are sorted alphabetically in each group of high-, middle- and low-income categories. The number of respondents (for countries with more than one respondent) is indicated in front of their names, and numbers in each column represent valid responses from each country.

### Statistical Analysis

Statistical analyses were performed using SPSS version 22 (IBM Corp., Armonk, NY, USA) and RStudio (version 1.2.1335). Descriptive data are presented as means and percentages for each country's response mean (percentage), as well as an average to the global responses.

### Ethics Approval

The survey protocols and all materials, including the survey questionnaires, received approval from the University of Social Welfare and Rehabilitation Sciences, an ethics committee in Tehran, Iran (Code: IR.USWR.REC.1399.061).

## Results

### Participants

**Figure 1:**
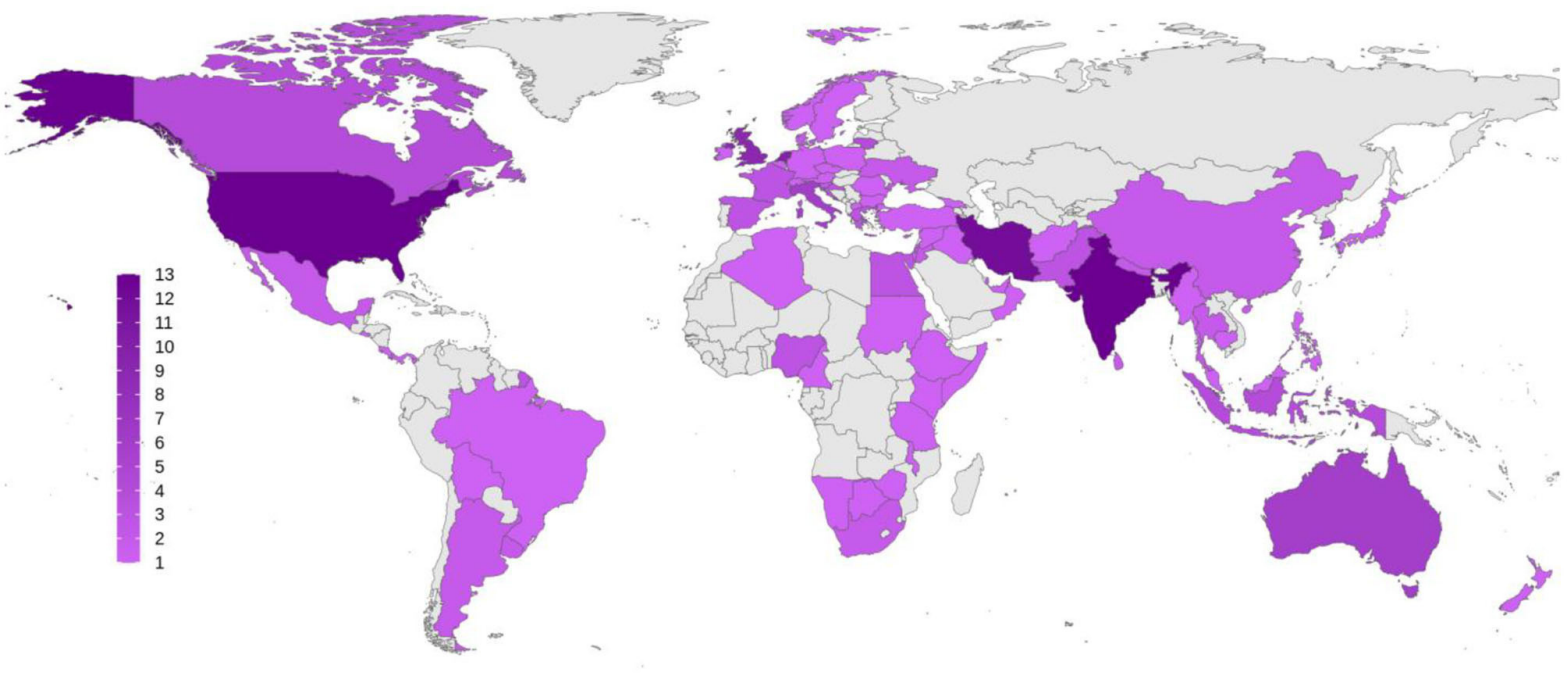
Global distribution of the respondents to the survey. Number of participants from each country is demonstrated as a color spectrum from light to dark purple.

A total of 177 respondents from 77 countries participated. Figure 1 shows the distribution of the countries and the number of participants from each. Among 177 respondents, 95 (53.7%) were from high-income, 34 (19.2%) from middle-income, and 48 (27.1%) from low-income countries (“World Economic Situation and Prospects 2019,” 2019). Table 1 shows respondents' demographic characteristics classified by their associated countries' income.

**Table 1:** Survey respondents' demographic, educational, and professional information classified by their countries' income status.

	**Total** ** (*n* = 177)**	**High-income** ** countries** ** (*n* = 95)**	**Middle-income** ** countries** ** (*n* = 34)**	**Low-income** ** countries** ** (*n* = 48)**
Age (year)	46.5 (10.8)	49.9 (10.1)	44.9 (8.2)	41.0 (11.2)
**Gender**				
Female	62 (35%)	39 (41.1%)	9 (26.5%)	14 (29.2%)
Male	111 (62.7%)	55 (57.9%)	23 (67.6%)	33 (68.8%)
Others	4 (2.3%)	1 (1.1%)	2 (5.8%)	1 (2.1%)
**Degree**				
BSc.	6 (3.4%)	4 (4.2%)	1 (2.9%)	1 (2.1%)
MSc	13 (7.3%)	2 (2.1%)	3 (8.8%)	8 (16.7%)
MD	72 (40.7%)	35 (36.8%)	11 (32.4%)	26 (54.2%)
PhD	31 (17.5%)	19 (20%)	9 (26.5%)	3 (6.2%)
MD, MSc	13 (7.3%)	9 (9.5%)	2 (5.9%)	2 (4.2%)
MD, PhD	32 (18.1%)	22 (23.2%)	5 (14.7%)	5 (10.4%)
Others	10 (5.6%)	4 (4.2%)	3 (8.8%)	3 (6.2%)
**Discipline**				
Addiction Medicine	19 (10.7%)	17 (17.9%)	0 (0%)	2 (4.2%)
Drug/Health Policy	8 (4.5%)	4 (4.2%)	1 (2.9%)	3 (6.2%)
General Medicine	17 (9.6%)	10 (10.5%)	6 (17.6%)	1 (2.1%)
Other Medical Specialties	3 (1.7%)	1 (1.1%)	1 (2.9%)	1 (2.1%)
Pharmacology	2 (1.1%)	2 (2.1%)	0 (0%)	0 (0%)
Psychiatry	95 (53.7%)	51 (53.7%)	13 (38.2%)	31 (64.6%)
Psychology/ Counseling	20 (11.3%)	8 (8.4%)	9 (26.5%)	3 (6.2%)
Social Work	5 (2.8%)	0 (0%)	3 (8.8%)	2 (4.2%)
Others	8 (4.5%)	2 (2.1%)	1 (2.9%)	5 (10.4%)

*Variables are reported as mean (standard deviation) or count (percent %)*.

*NA, not applicable; BSc, Bachelor of Science; MD, Doctor of Medicine; MSc, Master of Science; PhD, Doctor of Philosophy; Sig, significance; SD, standard deviation*.

### Implementing Business Continuity/Contingency Plans

**Figure 2:**
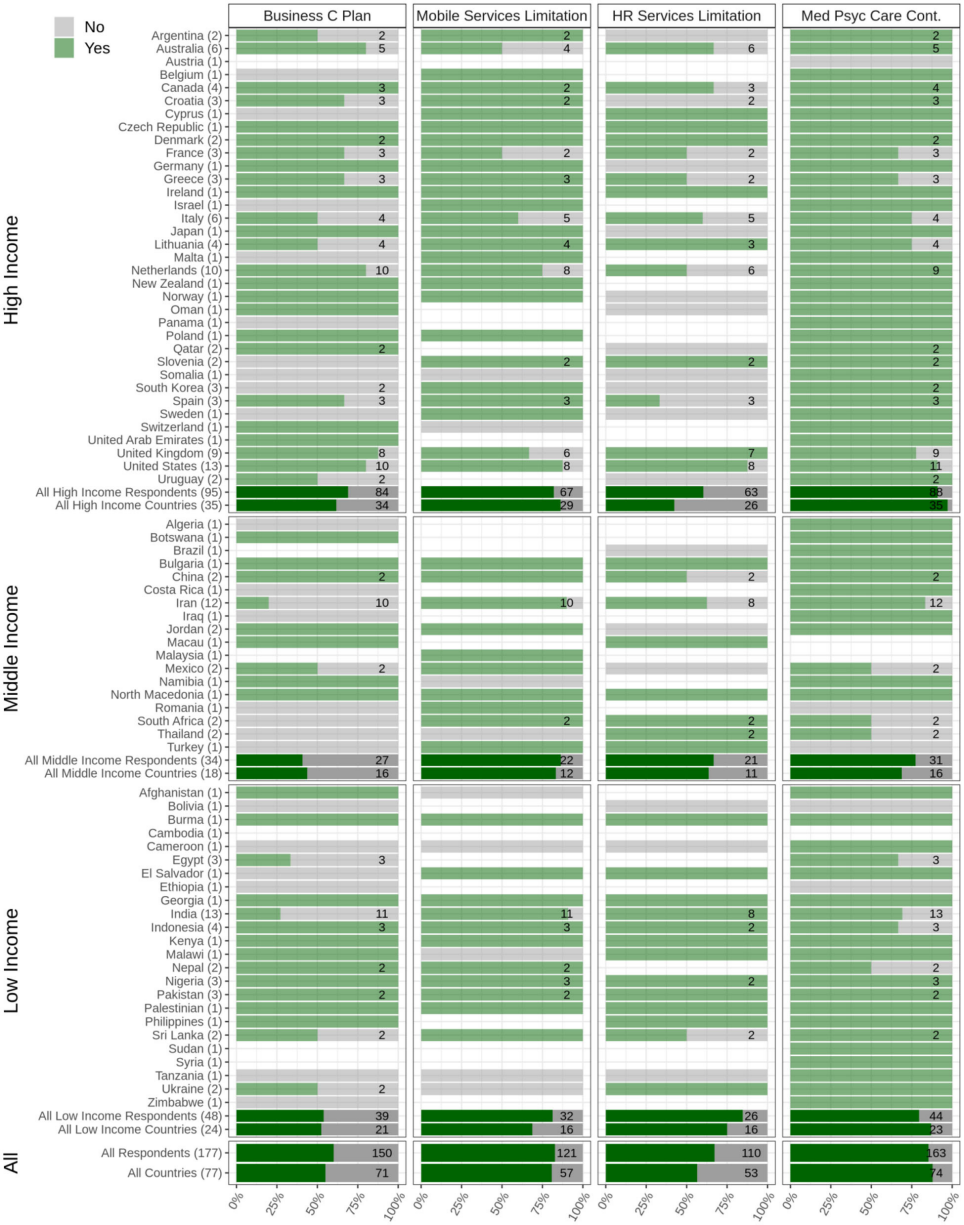
Availability and accessibility of treatment and harm reduction services. Data relating to arranging business continuity plans (Business C Plan), limitations that mobile services faced during the pandemic (mobile services limitation), limitations that harm reduction services faced during the pandemic (HR services limitation), and continuity of other medical and psychiatric necessary care (Med Psyc Care Cont.) are depicted. The Figure shows responses from respondents from 77 countries categorized into low-, middle-, and high-income countries. The light green bars and the numbers associated with each country show the survey respondents who reported having experienced limitations regarding the question in their country (Yes), and the gray bars show the survey respondents who reported having experienced no limitations regarding the question in their country (No). The dark green bars show the overall responses in each category (low, middle, and high income) as well as overall responses.

Among respondents from high-income countries (*N* = 95), 69% answered that business continuity/contingency plans had been implemented in their countries to make sure that services continued to operate for PWSUDs during the COVID-19 pandemic compared to 40.7% in the middle-income (*N* = 34) and 53.8% (*N* = 48) in low-income countries. Overall, respondents from 56% of participating countries reported that business contingency plans had been arranged to help ensure the continuity of services during the pandemic (Figure 2).

### Availability and Accessibility of Treatment and Harm Reduction Services

Among respondents from high-income countries (*N* = 95), 57% answered that treatment and harm reduction services for PWSUDs had been available and accessible in their countries during the pandemic onset compared to 51.6% in the middle-income (*N* = 34) and 63% in low-income (*N* = 48) countries. Overall, respondents from 59% of participating countries reported that treatment and harm reduction services for PWSUDs had been available and accessible during the initial period of the COVID-19 pandemic (Figure 2).

Respondents from over 81% of participating countries (*N* = 77) reported having experienced limitations in the usage of any outreach services due to lockdown policies for homeless PWSUDs. Furthermore, respondents from 57% of participating countries reported having experienced limitations in their harm reduction overdose services during the initial period of the pandemic. Problems with the distribution of take-home naloxone were reported by respondents from 57% of participating countries. Respondents from 54.8% of the participating countries reported shortages in needle and syringe programs (NSPs) and/or with respect to condom distribution.

### Medical and Psychiatric Care During the Initial Period of the Pandemic

**Figure 3:**
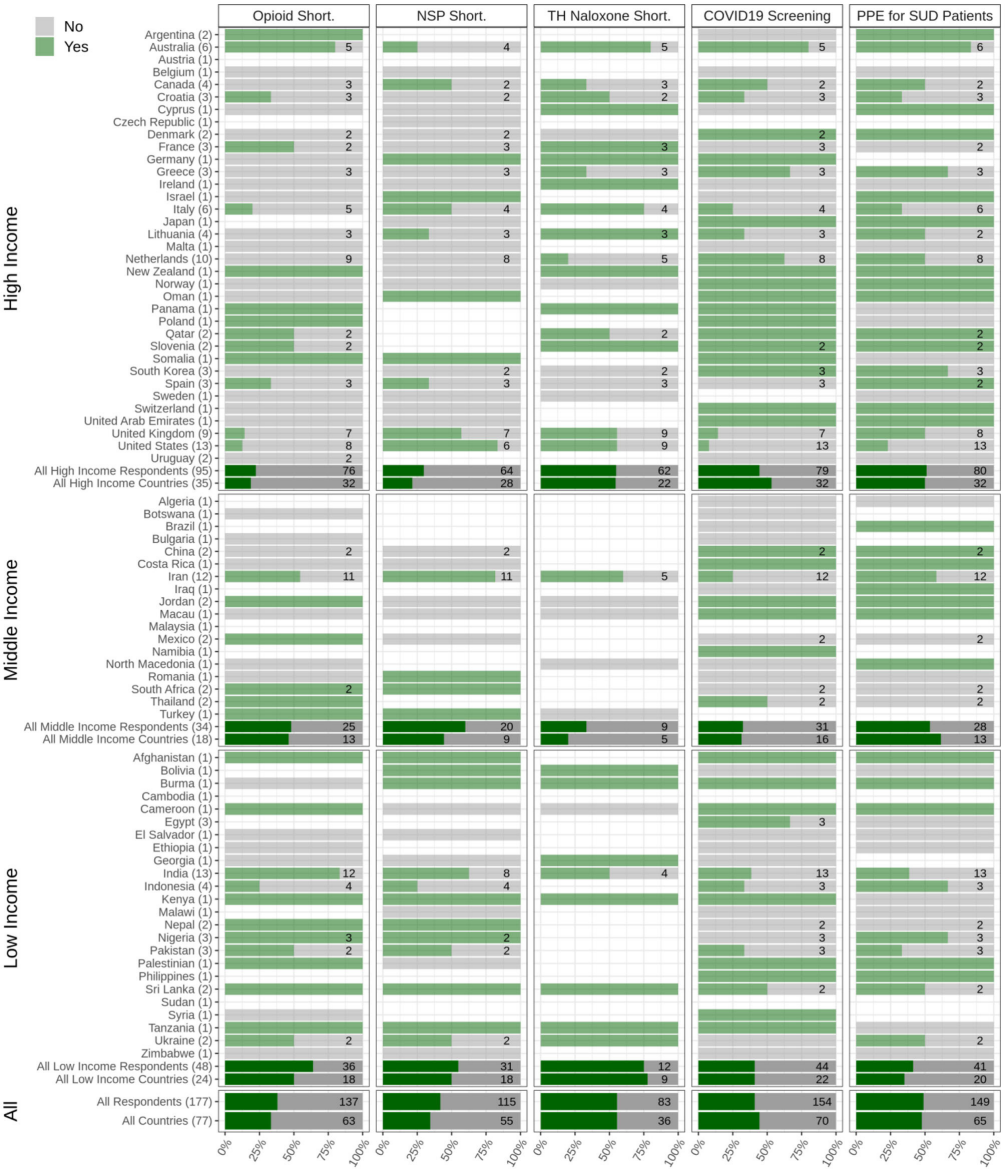
Medical services for people with substance use disorders during the pandemic. The responses of respondents from 77 countries are shown, categorized into low-, middle-, and high-income countries to the questions related to the shortages in opioid medication (opioid short.), disruption in needle and syringe and/or condom distribution services (NSP Short.), availability or shortages in take-home naloxone services (TH Naloxone short.), availability of COVID-19 screening kits and equipment for people with substance use disorders (PWSUDs) in their countries (COVID-19 screening), and personal protective equipment provision to PWSUDs (PPE for SUD patients).

Among respondents from high-income countries (*N* = 95), 90% answered that medical and psychiatric care for PWSUDs had been available during the initial stages of the pandemic compared to 77.4% in middle-income (*N* = 34) and 79.5% in low-income (*N* = 48) countries. Overall, respondents in 88% of participating countries reported that necessary medical and psychiatric care for PWSUDs had continued in their countries during this period (Figure 2). However, respondents in 37.5% of participating countries reported having experienced shortages of opioid medications (methadone or Buprenorphine) (Figure 3).

Only 44.3% of respondents from high-income (*N* = 95), 32.2% from middle-income (*N* = 34), and 40.1% from low-income (*N* = 48) countries reported that COVID-19 screening and/or diagnosis test kits based on local/national guidelines for PWSUD had been available in their country. Overall, respondents from only 48% of the participating countries reported that there had been enough personal protective equipment (PPE) available for PWSUDs during the initial stage of the pandemic. Respondents from 77.7% of participating countries reported SUD health workers' safety as a concern for employers in the outpatient treatment centers, 66.4% had received training regarding their safety, and 72.9% reported that they had had access to enough PPE (Figure 3).

**Figure 4:**
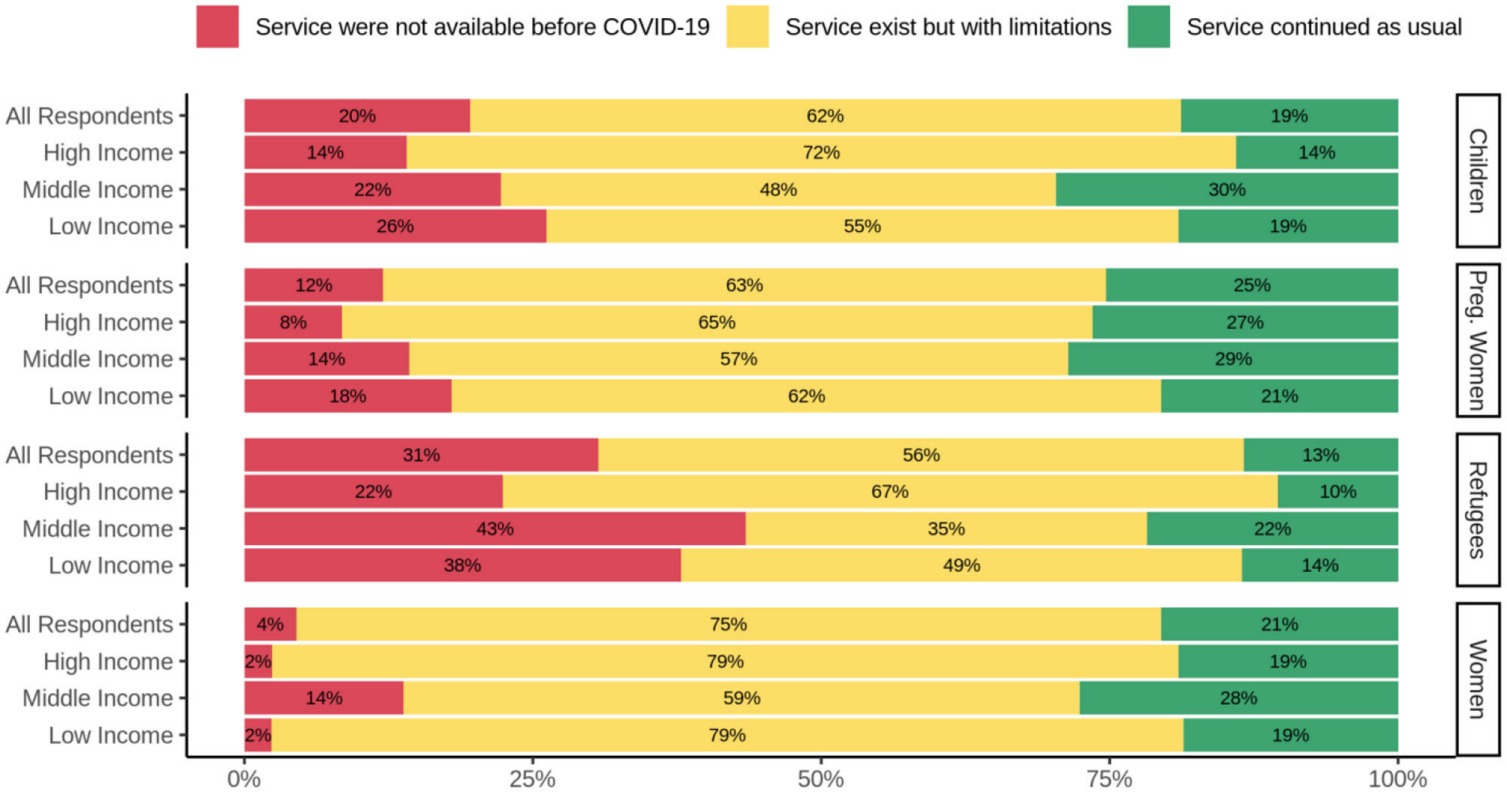
Effects of COVID-19 on substance use treatment and/or harm reduction services for vulnerable groups. Services for children, pregnant women, refugees, and women, in high-, middle-, and low-income countries are depicted. The red, yellow, and green bars depict the responses indicating lack of availability of services during the COVID-19 pandemic, the existence of limited services, and usual service provision, respectively.

The distribution of other responses on the effect of COVID-19 on substance use treatment and/or harm reduction services to vulnerable groups such as children, women, pregnant women, and immigrants or refugees can be seen in Table 2 and Figure 4. Table 2 shows the existence of services for children, women, pregnant women, and refugees or immigrants among the countries based on their income group.

**Table 2:** Services for children, women, pregnant women, and refugees or immigrants among the countries based on their income group.

**Target group**	**Total** ** % (*n*)**	**High income** ** countries** ** % (*n*)**	**Middle income** ** countries** ** % (*n*)**	**Low income** ** countries** ** % (*n*)**
**a. Service Availability**				
Children	80.8 (130)	79.4 (63)	92.3 (26)	75.6 (41)
Women	95.4 (153)	96.3 (81)	96.6 (30)	92.8 (42)
Pregnant Women	88 (149)	88.4 (78)	89.3 (28)	86) 43)
Immigrants/Refugees	70.1 (124)	68.2 (63)	82.6 (28)	65.8 (34)
**b. Continued as Usual**				
Children	22.8 (30)	18 (12)	16.6 (5)	35.5 (15)
Women	21 (33)	16.6 (14)	20.7 (6)	28.2 (12)
Pregnant Women	28.2 (42)	23.2 (18)	28 (8)	37.8 (16)
Immigrants/Refugees	18.4 (23)	11.6 (8)	21 (6)	28 (10)
**c. Continued with Limitations**				
Children	77.2 (100)	82 (51)	83.3 (21)	64.5 (26)
Women	79 (120)	77.2 (67)	83.4 (24)	79.3 (30)
Pregnant Women	71.8 (107)	76.8 (60)	72 (20)	62.2 (27)
Immigrants/Refugees	81.6 (101)	88.4 (55)	79 (22)	72 (24)

*Availability of the services is reported in Part a. Continuity of the service as usual or with limitations among countries that have the service available is reported in Parts b and c. Percent has been calculated based on Yes response in the respondents in each group of income*.

Overall, 22.8% of all respondents replied that service for children continued as usual compared to 77.2% that replied service for children continued but with limitations. According to the responses, in all three groups of income countries, treatment and/or harm reduction services for pregnant women were a group with minimum impact from COVID-19. Refugees and the immigrant population was the group that their services impacted more than other groups due to COVID-19. Only 18.4% replied that service for refugees and/or immigrants population continued as usual, and 81.6% replied that this service continued but with limitations.

### Health Policies for COVID-19 Among PWSUDs

Overall, respondents from 48% of the participating countries reported the presence of local and/or national guidelines tailored to be used during the initial stage of the pandemic (60.2% in high-income, 57.1% in middle-income, and 29% in low-income countries). Among respondents from high-income countries, 65.7% answered that there had been a protocol available for COVID-19 screening in various treatment sectors for PWSUDs or harm reduction facilities compared to 60% in middle-income and 82.3% in low-income countries.

Over 76% of respondents from high-income, 63.3% from middle-income, and 63% from low-income countries reported that there had been guidelines available that helped service providers in the management and/or referral of PWSUDs with symptoms of COVID-19.

Most respondents replied that there had been plans to restrict personal contacts and decrease patients' commutes for treatment in their countries (86, 90, and 86.6% in high-, middle- and low-income countries, respectively, and 85% overall) due to their national and regional lockdown policies.

**Figure 5:**
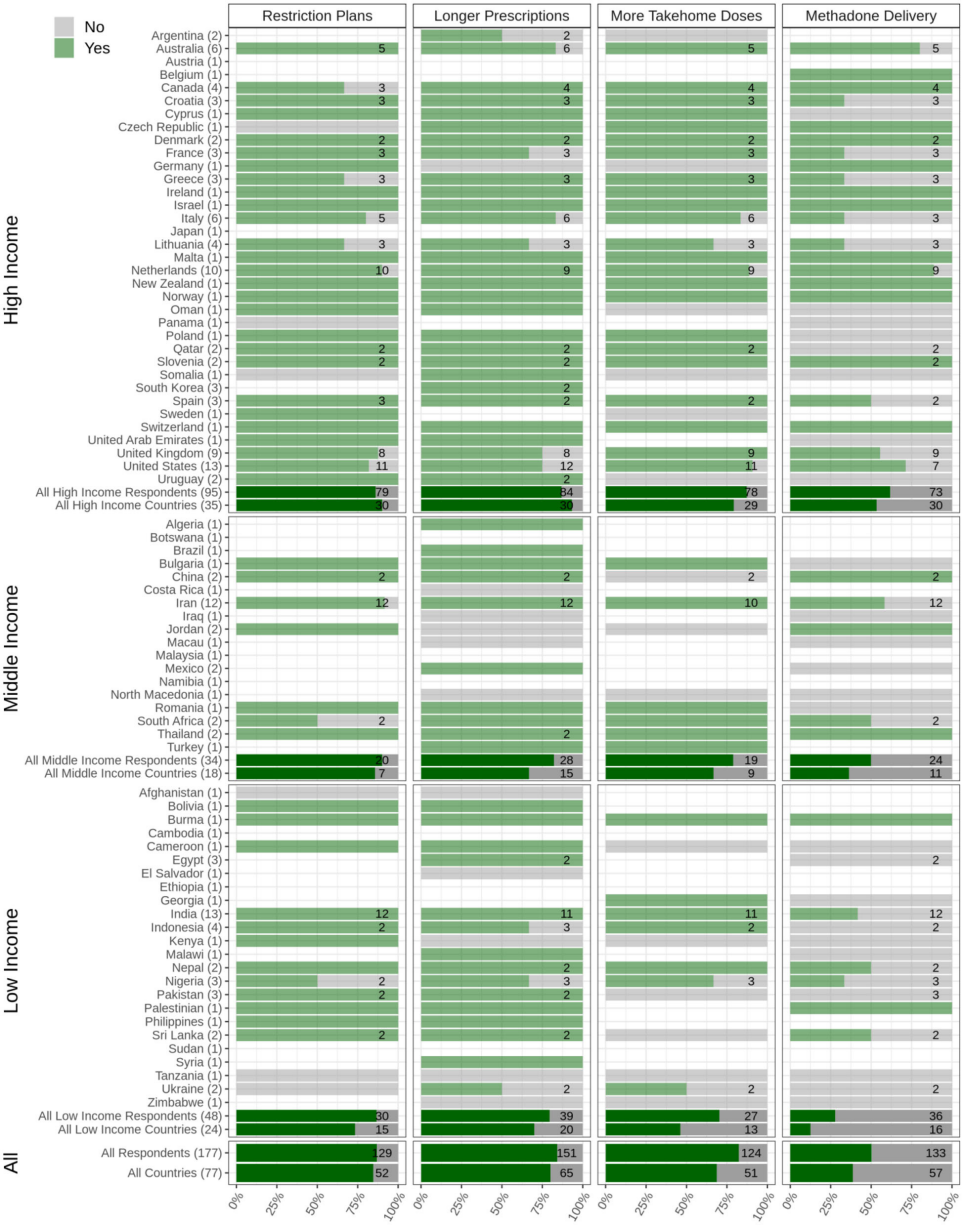
Health policies for COVID-19 among people with substance use disorders (PWSUDs). Plans to restrict any personal contact, provision of prescriptions of longer durations, provision of more take-home doses of opioids drugs, and availability of any program for delivering opioid drugs to patients' homes are depicted. The Figure shows responses from 77 countries, which are categorized into low, middle, and high income. The light green bars and the numbers associated with each country show the survey respondents who reported having experienced limitations regarding the question in their country (Yes), and the gray bars show the survey respondents who reported having experienced no limitations regarding the question in their country (No). The dark green bars show the overall responses in each category (low, middle, and high income) as well as overall responses.

As a result, respondents from 80% of the participating countries reported that clinicians had been prescribing longer-period prescriptions (e.g., 28 days rather than weekly) to PWSUDs during the onset of the pandemic (Figure 5).

Additionally, around 69% of participating countries reported that clinicians within OAT programs had provided more take-home doses of methadone and/or Buprenorphine during the onset of the pandemic. Regionally, 61.6% of respondents from high-income, 50% from middle-income, and 27.7% from low-income countries reported that this approach had been used in their countries (Figure 5).

Respondents from high-income countries most frequently reported having had the availability of long-acting injectable Buprenorphine (34.9%; *n* = 63). Overall, respondents from 22% of participating countries reported that long-acting injectable Buprenorphine had been available as a therapeutic option.

**Figure 6:**
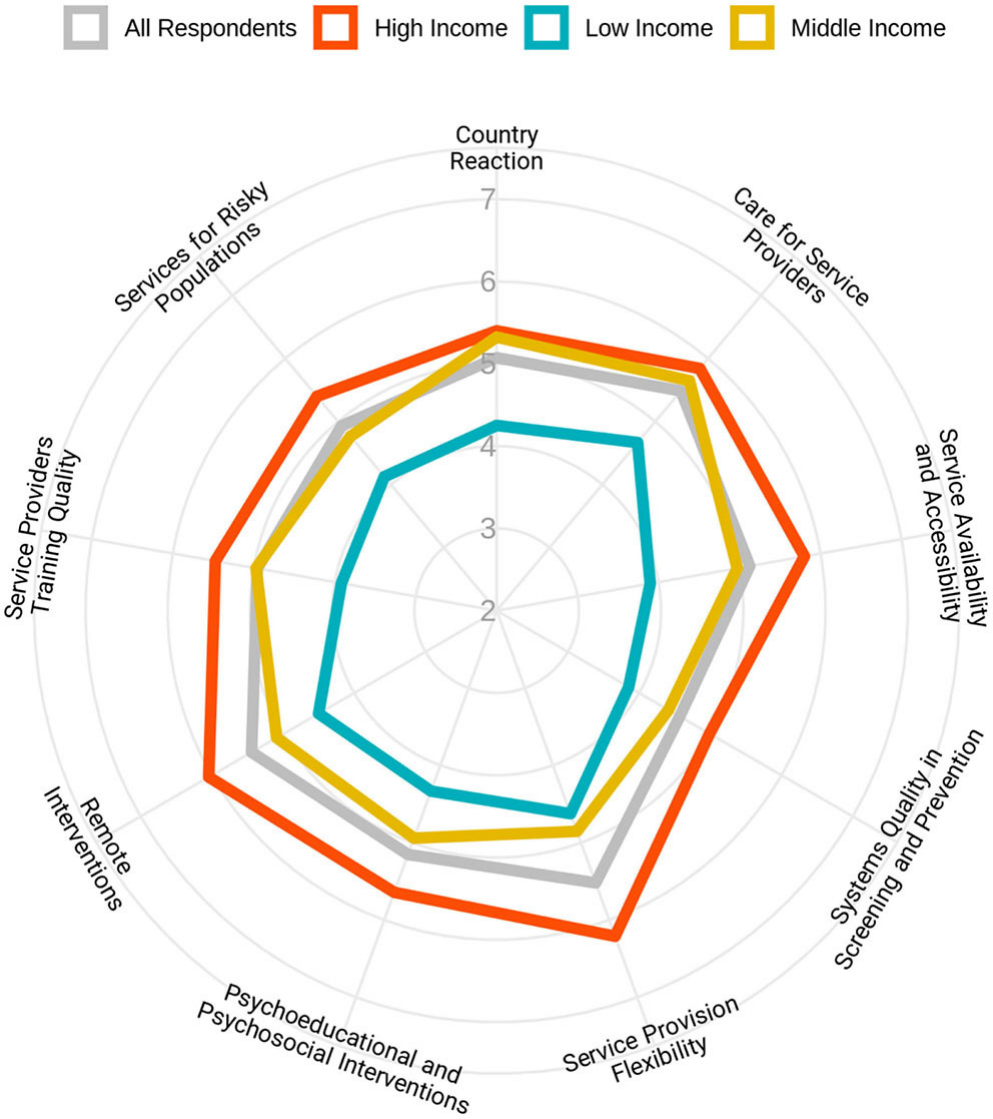
Flexibility of health responses for people with substance use disorders in response to the pandemic in different domains based on the income levels of the countries. Respondents were asked to rate the overall flexibility of their health system in nine different domains, from 1 (extremely poor) to 10 (extremely good).

Figure 6 shows the average score of each question based on income categorization. The maximum contrast between high- and low-income countries was seen in the availability and access to treatment and harm reduction services. Maximum and minimum differences between high- and middle-income countries were observed in flexibility in service provision and countries' reactions to the COVID-19 pandemic, respectively.

**Figure 7:**
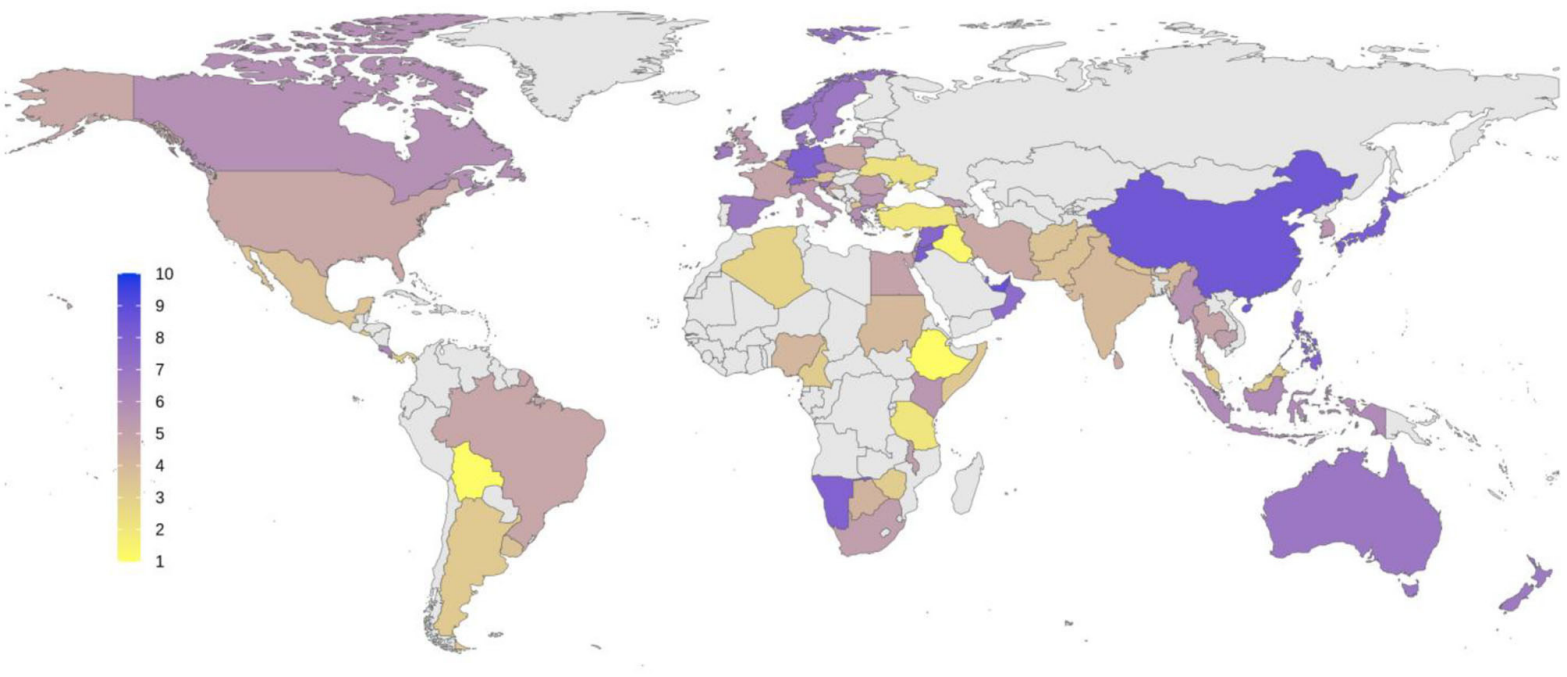
The overall quality of health response to COVID-19 pandemic based on the subjective ratings by respondents from different countries. Average scores were measured based on responses in nine domains depicted in Figure 6. Score 1 represents the worst quality in response, and 10 represents the best situation in favor of health services. Average scores for each country are shown using a color spectrum from yellow to blue.

An average for all rating scale questions in different domains has been calculated, and Figure 7 shows the results in a global map format.

## Discussion

The emergence of COVID-19 in early 2020 raised considerable challenges for substance use treatment and harm reduction programs worldwide, as reflected in this global survey. The need for effective spatial distancing and isolation to protect patients, the treatment workforce, and people in contact with patients and health workers have placed increased demands on treatment services provision, with potential imbalances in impact on particularly vulnerable patient populations (28). Here, in this global survey, we have explored different challenges and health responses in 77 countries. Our findings showed that respondents from 56% of participating countries reported business contingency plans had been arranged to help ensure that services would continue to operate during the pandemic, which is compatible with responses to another question indicating that 41% of respondents believed there had not been sufficient availability and accessibility of treatment and harm reduction services during the onset of the pandemic in their countries at the time of survey completion.

As a preventative measure to reduce COVID-19 spread, all international and national published guidelines advised limited but effective ways regarding how to initiate treatment, support stabilization, and maintenance and continue to provide harm reduction measures to treatment-seeking and other populations with substance use problems (4, 34). These recommendations often included extending flexibility in OAT services with reduced supervision of doses and increased home delivery (35). Another step taken to adjust to the present situation included expanding telemedicine and teletherapy services (5, 28, 34).

The COVID-19 pandemic is synergistically interacting with a substance use epidemic globally, creating a *syndemic* [defined as a synergistic epidemic, the aggregation of two or more concurrent or sequential epidemics, which exacerbate the prognosis and burden of disease (36)]. During the COVID-19 pandemic, marginalized people, including PWSUDs, are at greater risk of increased morbidity and mortality (37). These syndemic disadvantaged populations may be more likely to experience disparate, possibly substandard, service provision in systems prioritizing resource needs around a pandemic response (Inverse Response Law and Inverse Care Law) (38). Such inequities may present at macrolevels around effective and appropriate policymaking at national, organizational, and local levels (38) and at microlevels around areas of access to resources, social services, public health benefits of medical treatments, pharmacies, healthcare facilities, and provision of medical equipment (39).

Proactive business continuity plans for PWSUDs are important for all governments as part of COVID-19 remobilization plans and possible future responses to similar pandemics to support and avert delays and inequities in responses. Overall, PWSUDs are at risk for a negative impact of COVID-19 (6); it is also essential to mention that gender differences play a substantial role in the vulnerabilities of PWSUDs (40). Our findings showed that 88% of respondents reported continuity of other necessary medical and psychiatric care compared to <60% who reported the existence of business continuity/contingency plans and enough availability and accessibility of treatment and harm reduction services for PWSUDs. These findings suggest that policymakers and health authorities in each country could have possibly made more appropriate decisions in order to protect at-risk and marginalized PWSUDs including those who may be homeless, have HIV/AIDS, hepatitis, or multiple and complex morbidities. Such decisions may involve considering how to subtly provide scheduled and new appointments and prescription medications in the circumstances of lockdowns.

This study has multiple limitations that have been described in detail in the study protocol of the survey (31). The responses obtained were intentionally based around personal opinions of addiction medicine experts to help understand the “state of things in real life” rather than objective epidemiological data, which would have been considerably delayed. Therefore, ethical approval has not been taken from each of the countries that participated in the survey. The limited number of respondents makes this information non-representative and possibly biased. In other words, the survey results might be subject to bias and not demonstrate a true reflection of addiction services in their countries. Hence, the findings (opinions) have a high chance of subjective biasing. Sampling bias is another limitation, and indeed due to sampling methodology, the participants were not necessarily oriented to all domains of the questionnaire.

Given the urgency of the COVID-19 pandemic, the paper aims to alert and inform colleagues around the world and facilitate collaboration. Due to the time limitations, the questionnaire was circulated only in English. Therefore, some experts may have withdrawn from the survey for lingual reasons, and others may have answered questions less precisely.

## Conclusions

Based on our findings in this global survey, we conclude that the addiction medicine systems in all countries, regardless of income level, have been affected to some degree by the COVID-19 pandemic. Depending on the different domains and the ability of countries to adapt to existing conditions, these effects may differ across jurisdictions. Income level may relate importantly to responses and impact vulnerable groups like PWSUDs. Although this survey's findings should be interpreted with caution, the translation of our study results as recommendations for addiction medicine services, and policymakers would hopefully support a more resilient system of care that improves responses to future COVID-19 waves and other pandemics.

Continuity of services, especially in crises, needs certain evidence-based and locally tailored protocols and guidelines. In our study, addiction medicine professionals reported that most of their countries did not provide early guidelines or protocols to tailor their services to the pandemic. It is important to consider that respondents in only one-third of low-income countries reported the availability of such guidelines compared to respondents in half of the high-income countries. Another survey (41) conducted in four high-income regions (New South Wales, Ireland, Scotland, New York State, and British Columbia) found that special guidelines in response to the new situation and assurance of continuity of the services were available very soon after the start of lockdown, which is consistent with our findings that high-income countries had a more timely response in this domain. In the absence of guidelines and protocols, clinicians and service providers may not effectively balance various competing ethical and professional issues when they are making clinical and operational decisions when many things may be happening that could potentially be conflicting in nature (e.g., maintaining stability but reducing therapeutic contacts). Guidelines also allow stakeholders to improvise and identify innovative ways through evidence-based solutions to decrease the dual burden of substance use and COVID-19 infection (42). International organizations such as the WHO and United Nations Office of Drug Control (UNODC) and other related groups such as the International Society of Addiction Medicine (ISAM), International Society of Substance Use Professionals (ISSUP), and World Federation Against Drugs (WFAD) should provide adequate support to raise policymakers' knowledge in the area of addiction medicine on how to establish business continuity committees during initial stages of pandemics in order to make advanced care planning decisions through effective leadership.

Additionally, our results showed that respondents reported the shortage of opioid medication for maintenance treatment from about 40% of participating countries. Lack of opioid medications in patients undergoing maintenance treatment is a risk factor for a lapse, relapse, and/or overdoses. This situation may become more severe when transport and other supply chains are disrupted, compounded with the reduced provision by pharmacies and other dispensing outlets either due to spatial distancing, and reduced hours of service and/or closing during the pandemic.

According to this finding, we recommend that governments and local authorities be cognizant that an effective response system is based on a well-informed and supportive environment. Available and communicated international and national clinical guidelines are pivotal in future responses to similar pandemics when supporting PWSUDs.

The World Drug Report 2020 stated that “*If Governments respond the same way to the current economic slump, interventions such as prevention of drug use and related risk behaviors and drug treatment services could be hard hit*” (43). Substance use accounts for ~11% of the global health burden (44). Treatment is a critical strategy for reducing the burden of the disease. A study of World Mental Health Surveys (45) found that only 7.1% of PWSUDs had received at least minimally adequate treatment in the past year (10.3, 4.3, and 1.0%, respectively, in high-, upper-middle, and low/lower-middle-income countries) (46). Poor access to treatment, awareness/perceived treatment need, and compliance (on the part of both provider and client) have been reported to be the main barriers to substance use treatment (46).

Our results also show that harm reduction services seem to be among the most affected during the initial stages of the COVID-19 pandemic. Eighty-one percent of participating countries reported limitations in usage of any mobile and other outreach services due to lockdown policies for homeless PWSUDs, with respondents from 57% of participating countries reporting limitations in their harm reduction overdose services during the initial period of the pandemic. This was compounded with reported problems with the distribution of take-home naloxone as reported by respondents from 57% of participating countries. Finally, respondents from 54.8% of participating countries reported that there had been shortages at needle and syringe programs and/or of condom distribution. International organizations with regional and local government structures should create contingencies around adequate supplies of medications such as methadone and Buprenorphine. Harm reduction services, especially outreach services, are among the most effective strategies for preventing HIV, hepatitis C virus (HCV), and hepatitis B virus (HCV) transmission among the most at-risk populations (47).

Pregnant women and immigrants/refugees with SUDs are particularly among vulnerable groups. According to our survey responses, pregnant women were perceived as relatively less impacted during the initial period of the pandemic. This is reassuring, as discontinuity of treatment services could place not only a pregnant woman at high risk but also the developing fetus. However, refugee and immigrant populations were reported as having had their services impacted more than other groups due to the pandemic. Only 12.9% of respondents replied that service for refugees and/or immigrants population continued as usual, and 57.3% replied that this service continued but with severe limitations (48).

These findings highlight the fact that harm reduction initiatives should be seen as an integral part of an evidence-based treatment program and not as an adjunct to failed treatment and/or solely as a public health response to reduce blood-borne diseases. Service providers should be considering identifying person-centered, continuous care provision in all therapeutic options available (harm reduction initiatives included), especially during pandemic situations.

Lastly, our findings suggest that, in general, in multiple domains of countries' reactions to the pandemic (e.g., availability of and access to treatment and harm reduction, screening and early interventions, flexibility in service provision and services for special and high-risk populations), the COVID-19 pandemic has had a more negative impact that is linked to the income level of countries. Vulnerable groups such as immigrants and refugees with SUDs should have access to all possible therapeutic options available as described in the UN charter in the Human Rights Convention (“International Convention on the Protection of the Rights of All Migrant Workers and Members of Their Families”). Appropriate evidence-based services must be designed and implemented by health authorities for such vulnerable groups. Availability of all relevant resources is essential in the delivery of quality services.

## Declarations

Due to the methodological limitations of the study, the findings of this survey might not demonstrate the exact situation of the countries. AB is a staff member of UNODC. The authors alone are responsible for the views expressed in this article, and they do not necessarily represent the decisions or policies of the UNODC or other organizations.

## Data Availability Statement

The raw data supporting the conclusions of this article will be made available by the authors upon request.

## Ethics Statement

The studies involving human participants were reviewed and approved by The University of Social Welfare and Rehabilitation Sciences ethics committee, Tehran, Iran (Code: IR.USWR.REC.1399.061). The patients/participants provided their written informed consent to participate in this study.

## Author Contributions

SRR, AF, HE, CD, and AMB conceived and designed the study. SRR, AF, PR, MV, HE, CD, and AMB conducted the survey and collected the data. ME and PR analyzed the data and ran the statistical analyses. SRR, AF, HE, CD, MY, and AMB supervised the analysis and gave conceptual advice. SRR, AF, PR, MV, HE, CD, and AMB contributed to drafting the manuscript. CK, SA, AB, and MP edited the manuscript. All authors discussed the results and implications and commented on the final manuscript.

## ISAM-PPIG Global Survey Consortium Members

Adrian Octavian Abagiu^1^, Franck David Noel Abouna^2^, Mohamed Hassan Ahmed^3^, Basma Al-ansari^4^, Feda Mahmmoud Abu Al-khair^5^, Mandhar Humaid Almaqbali^6^, Atul Ambekar^7^, Hossein Mohaddes Ardabili^8^, Sidharth Arya^9^, Victor Olufolahan Lasebikan^10^, Murad Ali Ayasreh^11^, Debasish Basu^12^, Zoubir Benmebarek^13^, Roshan Bhad^7^, Mario Blaise^14^, Nicolas Bonnet^15^, Jennifer Brasch^16^, Barbara Broers^17^, Jenna L. Butner^18^, Moses Camilleri^19^, Giovanna Campello ^20^, Giuseppe Carra^21^, Ivan Celic^22^, Fatemeh Chalabianloo^23^, Abhishek Chaturvedi^24^, José de Jesús Eduardo Noyola Cherpitel^25^, Kelly J. Clark^26^, Melissa Anne Cyders^27^, Ernesto de Bernardis^28^, John Edward Derry^29^, Naveen Kumar Dhagudu^30^, Pavla Dolezalova^31^, Geert Dom^32^, Adrian John Dunlop^33^, Mahmoud Mamdouh Elhabiby^34^, Hussien Elkholy^35^, Nsidibe Francis Essien^36^, Ghandi Ilias Farah^37^, Marica Ferri^38^, Georgios D Floros^39^, Catherine Friedman^40^, Clara Hidalgo Fuderanan^41^, Gilberto Gerra^17^, Abhishek Ghosh^12^, Maka Gogia^42^, Ilias A. Grammatikopoulos^43^, Paolo Grandinetti^44^, Amira Guirguis^45^, David Gutnisky^46^, Paul Steven Haber^47^, Peyman Hassani-Abharian^48^, Zahra Hooshyari^49^, Islam Ibrahim Mokhtar Ibrahim^34^, Hada Fong-ha Ieong^50^, Regina Nova Indradewi^51^, Shelly Iskandar^52^, Shobhit Jain^53^, Sandi James^54^, Seyyed Mohammad hossein Javadi^55^, Keun Ho Joe^56^, Darius Jokubonis^57^, Acka Tushevska Jovanova^58^, Rama Mohamed Kamal^59^, Alexander Ivanov Kantchelov^60^, Preethy Kathiresan^7^, Gary Katzman^61^, Paul Kawale^62^, Audrey Margaret Kern^63^, Felix Henrique Paim Kessler^64^, Sung-Gon Sue Kim^65^, Ann Marie Kimball^66^, Zeljko Kljucevic^67^, Kristiana Siste^68^, Roneet Lev^69^, Hae Kook Lee^70^, Aiste Lengvenyte^71^, Shaul Lev-ran^72, 73^, Geni Seseja Mabelya^74^, Mohamed Ali El Mahi^75^, J. Maphisa Maphisa^76^, Icro Maremmani^77^, Laura Masferrer^78^, Orlagh McCambridge^79^, Garrett Gregory McGovern^80^, Aung Kyi Min^81^, Amir Moghanibashi-Mansourieh^56^, Jazman Mora-Rios^82^, Indika Udaya Kumara Mudalige^83^, Diptadhi Mukherjee^84^, Pejic Munira Munira^85^, Bronwyn Myers^86^, Jayakrishnan Menon T N^87^, Venkata Lakshmi Narasimha^88^, Nkemakolam Ndionuka^89^, Ali-Akbar Nejatisafa^90^, Kamran Niaz^20^, Asad Tamizuddin Nizami^91^, Jan H. Nuijens^92^, Laura Orsolini^93^, Vantheara Oum^94^, Adegboyega Adekunle Oyemade^95^, Irena Rojnia Palavra^96^, Sagun Ballav Pant^97^, Joselyn Paredes^98^, Eric Peyron^99^, Randall Alberto Quirós^100^, Rouhollah Qurishi^101^, Noor ul Zaman Rafiq^102^, Ranjini Raghavendra Rao^103^, Woraphat Ratta-apha^104^, Karren-Lee Raymond^105^, Jens Reimer^106^, Eduardo Renaldo^107^, Tara Rezapour^108^, James Roy Robertson^109^, Carlos Roncero^110^, Fazle Roub^111^, Elizabeth Jane Rubenstein^112^, Claudia Ines Rupp^113^, Elizabeth Saenz^20^, Mohammad Salehi^114^, Lampros Samartzis^115^, Laura Beatriz Sarubbo^116^, Nusa Segrec^117^, Bigya Shah^118^, Hongxian Shen^119^, Tomohiro Shirasaka^120^, Steve Shoptaw^121^, Fransiskus Muronga Sintango^122^, Veronica Andrea Sosa^123^, Emilis Subata^124^, Norberto Sztycberg^125^, Fatemeh Taghizadeh^126^, Joseph Brian Tay Wee Teck^127^, Christian Tjagvad^128^, Marta Torrens^129^, Judith Meme Twala^130^, Ramyadarshni Vadivel^131^, Joseph Robert Volpicelli^132^, Jelmer Weijs^133^, Steven Michael Wintoniw^134^, Apisak Wittayanookulluk^135^, Marcin Wojnar^136^, Sadia Yasir^91^, Yimenu Yitayih^137^, Min Zhao^138^ and Arash Khojasteh Zonoozi^139^

^1^ Prof. Dr. Matei Bals- Arena OMT Department, National Institute for Infectious Diseases, Bucharest, Romania

^2^ Faculty of Medicine and Biomedical Sciences, University of Yaoundé 1, Yaoundé, Cameroon

^3^ Alamal psychiatric hospital, Dubai, United Arab Emirates

^4^ Sydney Medical School, University of Sydney, Sydney, NSW, Australia

^5^ Al Ahliyya Amman University, Amman, Jordan

^6^ Ministry of Health, Muscat, Oman

^7^ Department of Psychiatry and National Drug Dependence Treatment Center (NDDTC), All India Institute of Medical Sciences (AIIMS), New Delhi, India

^8^ Faculty of Medicine, Psychiatry and Behavioral Sciences Research Center, Ibn-e-Sina Hospital, Mashhad University of Medical Sciences, Mashhad, Iran

^9^ State Drug Dependence Treatment Center, Institute of Mental Health, Pt BDS University of Health Sciences, Rohtak, India

^10^ Department of Psychiatry, College of Medicine, University of Ibadan, Ibadan, Nigeria

^11^ Addiction medicine clinic, Amman, Jordan

^12^ Department of Psychiatry, Drug De-addiction & Treatment Center, Postgraduate Institute of Medical Education & Research, Chandigarh, India

^13^ Addiction medicine clinic, Mila, Algeria

^14^ Center medical Marmottan, Paris, France

^15^ Réseau de Prévention des Addictions (RESPADD), Paris, France

^16^ Department of Psychiatry and Behavioral Neurosciences, Michael DeGroote School of Medicine, McMaster University, Hamilton, ON, Canada

^17^ Geneva University Hospitals, Geneva, Switzerland

^18^ CUNY School of Medicine, New York, NY, United States

^19^ Aġenzija Sedqa, Santa Venera, Malta

^20^ United Nations Office on Drugs and Crime (UNODC), Vienna, Austria

^21^ Department of Medicine and Surgery, University Milan-Bicocca, Milan, Italy

^22^ University Psychiatric Hospital Vrapce, Zagreb, Croatia

^23^ Department of Addiction Medicine, Haukeland University Hospital, Bergen, Norway

^24^ Department of Biochemistry, Melaka Manipal Medical College, Manipal Academy of Higher Education, Manipal, India

^25^ Addiction medicine clinic, Mexico City, Mexico

^26^ Addiction Crisis Solutions, Louisville, KY, United States

^27^ Department of Psychology, Indiana University Purdue University - Indianapolis, Indianapolis, IN, United States

^28^ SerT Lentini, ASP Siracusa, Syracuse, Italy

^29^ Serenity Vista Addiction Treatment Center, Jaramillo, Panama

^30^ Department of Psychiatry, ESIC Medical College, Hyderabad, India

^31^ National Institute of Mental Health, Klecany, Czechia

^32^ Collaborative Antwerp Psychiatric Research Institute (CAPRI), Antwerp University (UA), Antwerp, Belgium

^33^ Drug & Alcohol Clinical Services, Hunter New England Local Health District, New Lambton, NSW, Australia

^34^ Ain Shams University, Cairo, Egypt

^35^ Department of Neurology and Psychiatry, Faculty of Medicine, Ain Shams University, Cairo, Egypt

^36^ Center for Research and Information on Substance Abuse, Jos, Nigeria

^37^ Addiction Medicine Clinic, Damascus, Syria

^38^ European Monitoring Center for Drugs and Drug Addiction (EMCDDA), Lisbon, Portugal

^39^ 2nd Department of Psychiatry, Aristotle University of Thessaloniki, Thessaloniki, Greece

^40^ Brown University and Lifespan Health System, Providence, RI, United States

^41^ Fuderanan Mental Health Clinic, Manila, Philippines

^42^ Georgian Harm Reduction Network, Tbilisi, Georgia

^43^ Organization Against Drugs, Primary Care Health Center, Veria, Greece

^44^ Addictions Services (Ser.D.), Department of Territorial Assistance, ASL Teramo, Teramo, Italy

^45^ Swansea University Medical School, Institute of Life Sciences 2, Sketty, United Kingdom

^46^ Hospital Borda, Universidad de Buenos Aires, Buenos Aires, Argentina

^47^ University of Sydney, Sydney, NSW, Australia

^48^ Institutes for Cognitive Science Studies (IRICSS), Brain and Cognition Clinic, Tehran, Iran

^49^ Tehran University of Medical Sciences, Tehran, Iran

^50^ Department of Anesthesiology, Yale University, New Haven, CT, United States

^51^ Drugs Rehabilitation Center, National Narcotics Board of Indonesia, East Jakarta, Indonesia

^52^ Department of Psychiatry, Universitas Padjadjaran, Bandung, Indonesia

^53^ Department of Psychiatry, Heritage Institute of Medical Sciences (HIMS), Varanasi, India

^54^ Univeristi Malaysia Sabah, Sabah, Malaysia

^55^ Department of Social Work, University of Social Welfare & Rehabilitation Sciences, Tehran, Iran

^56^ National Center for Mental Health of Korea, Seoul, South Korea

^57^ Republican Center for Addictive Disorders, Vilnius, Lithuania

^58^ Addiction medicine clinic, Skopje, North Macedonia

^59^ Naufar Institute, Doha, Qatar

^60^ The Kantchelov Clinic, Sofia, Bulgaria

^61^ Mount Sinai Medical Center, New York, NY, United States

^62^ African Institute for Development Policy, Lilongwe, Malawi

^63^ Sobriety Centers of New Hampshire, Antrim, NH, United States

^64^ Federal University of Rio Grande do Sul, Porto Alegre, Brazil

^65^ Department of Neuropsychiatry, Pusan National University Yangsan Hospital, YangsanSouth Korea

^66^ Chatham House, Washington, United States

^67^ Institute for Public Health of Split-Dalmatia County, Split, Croatia

^68^ Faculty of Medicine, Universitas Indonesia-Ciptomangunkusumo Hospital, Jakarta, Indonesia

^69^ Scripps Mercy Hospital, San Diego, CA, United States

^70^ Department of Psychiatry, The Catholic University of Korea, Seoul, South Korea

^71^ Faculty of Medicine, Institute of Clinical Medicine, Psychiatric Clinic, Vilnius University, Vilnius, Lithuania

^72^ Tel Aviv University, Tel Aviv, Israel

^73^ Israel Center on Addiction, Netanya, Israel

^74^ Community Health Work, Dar es Salaam, Tanzania

^75^ Hayat Center for Treatment and Psycho-social Rehabilitation, Khartoum, Sudan

^76^ University of Botswana, Gaborone, Botswana

^77^ V.P. Dole, Dual Disorder Unit, Santa Chiara University Hospital, University of Pisa, Pisa, Italy

^78^ CAS Girona, Department of Psychology, University of Girona, Girona, Spain

^79^ Community addiction team, Southern Health and Social Care Trust, Craigavon, United Kingdom

^80^ Priority Medical Clinic, Dublin, Ireland

^81^ Save the Children International, Yangon, Myanmar

^82^ Dirección de Investigaciones Epidemiológicas y Sociales, Instituto Nacional de Psiquiatría Ramón de la Fuente Muñiz, Mexico City, México

^83^ Department of Psychiatry, Faculty of Medicine, Sir John Kotelawala Defense University, Colombo, Sri Lanka

^84^ Center for Addiction Medicine, NIMHANS, Bangalore, India

^85^ Kleopatra Kodric, Irena Nisic, Ljubljana, Slovenia

^86^ Alcohol Tobacco and Other Drug Research Unit, South African Medical Research Council, Cape Town, South Africa

^87^ NIMHANS, Bangalore, India

^88^ Department of Psychiatry, Center for Addiction Medicine, National Institute of Mental Health and Neurosciences, Bengaluru, India

^89^ Federal Neuropsychiatric Hospital, Calabar, Nigeria

^90^ Department of Psychiatry, Psychosomatic Research Center, Tehran University of Medical Sciences, Tehran, Iran

^91^ Institute of Psychiatry, WHO Collaborating Center for Mental Health, Rawalpindi, Pakistan

^92^ Brijder Addiction Care, Zaandam, Netherlands

^93^ Department of Clinical Neurosciences/DIMSC, Unit of Clinical Psychiatry, School of Medicine, Polytechnic University of Marche, Ancona, Italy

^94^ Koh Kong Provincial Hospital, Phoumin, Cambodia

^95^ Kaiser Permanente, Oakland, CA, United States

^96^ Psychiatric hospital Sveti Ivan, Zagreb, Croatia

^97^ Department of Psychiatry and mental health, Institute of Medicine, Tribhuvan University, Kirtipur, Nepal

^98^ Universidad de El Salvador, San Salvador, El Salvador

^99^ AddiPsy, Lyon, France

^100^ Addiction medicine clinic, San José, Costa Rica

^101^ Novadic-Kentron Addiction Care Network, Vught, Netherlands

^102^ Phoenix Foundation for Research and Development, Lahore, Pakistan

^103^ Barwon Health, Geelong, VIC, Australia

^104^ Faculty of Medicine Siriraj Hospital, Mahidol University, Salaya, Thailand

^105^ University of the Sunshine Coast (USC), Queensland, QLD, Australia

^106^ Center for Interdisciplinary Addiction Research, University Medical Center Hamburg-Eppendorf, Hamburg, Germany

^107^ Drugs Rehabilitation Center, National Narcotics Board of Indonesia, East Jakarta, Indonesia

^108^ Department of Cognitive Psychology, Institute for Cognitive Science Studies, Tehran, Iran

^109^ Usher Institute, University of Edinburgh, Edinburgh, United Kingdom

^110^ Psychiatry Service, University of Salamanca Health Care Complex, School of Medicine, University of Salamanca, Salamanca, Spain

^111^ PGIMER, Chandigarh, India

^112^ Street Health Center, Kingston, ON, Canada

^113^ Department of Psychiatry, Psychotherapy, and Psychosomatics, Medical University Innsbruck, Innsbruck, Austria

^114^ Department of Neurosciences and Addiction Studies, School of Advanced Technologies in Medicine, Tehran University of Medical Sciences, Tehran, Iran

^115^ Medical School, University of Cyprus, Cyprus

^116^ Clínica Psiquiátrica de la Facultad de Medicina, Uruguay

^117^ Center for Treatment of Drug addiction, University Psychiatric Clinic, Ljubljana, Slovenia

^118^ Department of Psychiatry, Patan Academy of Health Sciences, School of Medicine, Lagankhel, Nepal

^119^ Department of Psychiatry, Second Xiangya Hospital, Central South University, Changsha, China

^120^ Department of Psychiatry, Teine Keijinkai Medical Center, Sapporo, Hokkaido, Japan

^121^ Department of Family Medicine, David Geffen School of Medicine at UCLA, Los Angeles, CA, United States

^122^ Health Professions Councils of Namibia, Windhoek, Namibia

^123^ Addiction medicine clinic, Montevideo, Uruguay

^124^ Republican Center for Addictive Disorders, Vilnius, Lithuania

^125^ Asociasion Programa Andres Argentina, Santa Fe, Argentina

^126^ Mazandaran University of Medical Sciences, Mazandaran, Iran

^127^ MRC/CSO SPHSU, University of Glasgow, Glasgow, United Kingdom

^128^ Gladsaxe Substance Use Disorder Treatment Center, Gladsaxe, Denmark

^129^ Institut de Neuropsiquiatria i Addiccions, IMIM-Hospital del Mar, Medical Research Barcelona, Spain

^130^ NACADA, Nairobi, Kenya

^131^ Waikato District Health Board (WDHB) Hamilton, Hamilton, New Zealand

^132^ Institute of Addiction Medicine, Plymouth Meeting, PA, United States

^133^ Jellinek, Amsterdam, Netherlands

^134^ Addictions Foundation of Manitoba, Manitoba, Canada

^135^ Thanyarak Chiangmai hospital, Khilek, Thailand

^136^ Medical University of Warsaw, Warsaw, Poland

^137^ Jimma University, Jimma, Ethiopia

^138^ Shanghai Mental Health Center, Shanghai Jiao Tong University School of Medicine, Shanghai, China

^139^ Student Research Committee, Faculty of Medicine, Mashhad University of Medical Sciences, Mashhad, Iran

## Conflict of Interest

The authors declare that the research was conducted in the absence of any commercial or financial relationships that could be construed as a potential conflict of interest.
